# DART-bid (Dose-differentiated accelerated radiation therapy, 1.8 Gy twice daily)–a novel approach for non-resected NSCLC: final results of a prospective study, correlating radiation dose to tumor volume

**DOI:** 10.1186/1748-717X-8-49

**Published:** 2013-03-05

**Authors:** Karl Wurstbauer, Heinz Deutschmann, Karin Dagn, Peter Kopp, Franz Zehentmayr, Bernd Lamprecht, Peter Porsch, Birgit Wegleitner, Michael Studnicka, Felix Sedlmayer

**Affiliations:** 1 Department of Radiation Oncology and radART-Institute for research and development on Advanced Radiation Technologies, Paracelsus Medical University, Salzburg, Austria; 2 Department of Pneumology, Paracelsus Medical University, Salzburg, Austria; 3 Universitätsklinik für Radiotherapie, Müllner Hauptstrasse 48, Salzburg, A-5020, Austria

**Keywords:** Lung cancer, Non-small cell lung cancer, Accelerated radiotherapy, Conformal radiotherapy, Target splitting, Prospective clinical trials, Combined modality, Treatment time, Accelerated repopulation, DART-bid

## Abstract

**Background:**

Sequential chemo-radiotherapies with intensive radiation components deliver promising results in non-resected non-small cell lung cancer (NSCLC). In general, radiation doses are determined by dose constraints for normal tissues, not by features relevant for tumor control. DART-bid targets directly the doses required for tumor control, correlating doses to tumor volume in a differentiated mode.

**Materials/Methods:**

Radiation doses to primary tumors were aligned along increasing tumor size within 4 groups (<2.5 cm/2.5–4.5 cm/4.5–6.0 cm/>6.0 cm; mean number of three perpendicular diameters). ICRU-doses of 73.8 Gy/79.2 Gy/84.6 Gy/90.0 Gy, respectively, were applied. Macroscopically involved nodes were treated with a median dose of 59.4 Gy, nodal sites about 6 cm cranial to involved nodes electively with 45 Gy. Fractional doses were 1.8 Gy twice daily (bid).

2 cycles chemotherapy were given before radiotherapy.

Between 2004 and 2009, 160 not selected patients with 164 histologically/cytologically proven NSCLC were enrolled; Stage I: 38 patients; II: 6 pts.; IIIA: 69 pts.; IIIB: 47 pts. Weight loss >5%/3 months: 38 patients (24%).

Primary endpoints are local and regional tumor control rates at 2 years (as >90% of locoregional failures occur within 2 years). Secondary endpoints are survival and toxicity. With a minimum follow-up time of 2 years for patients alive, the final results are presented.

**Results:**

32 local and 10 regional recurrences occurred. The local and regional tumor control rates at 2 years are 77% and 93%, respectively.

The median overall survival (OS) time is 28.0 months, the 2- and 5-year OS rates are 57% and 19%, respectively. For stage III patients, median OS amounts to 24.3 months, 2- /5-year OS rates to 51% and 18%, respectively.

2 treatment-related deaths (progressive pulmonary fibrosis) occurred in patients with pre-existing pulmonary fibrosis. Further acute and late toxicity was mild.

**Conclusions:**

This novel approach yields a high level of locoregional tumor control and survival times. In general it is well tolerated. In all outcome parameters it seems to compare favourably with simultaneous chemo-radiotherapies, at present considered ‘state of the art’; and is additionally amenable for an unselected patient population.

## Introduction

Lung cancer is the most commonly found malignant disease worldwide, and the leading cause of death due to cancer. About 80 percent of lung cancer patients are affected by tumors with non-small cell histologies (NSCLC); and 30–40% of them at diagnosis have locoregionally limited, but-for tumor extension or medical reasons-inoperable diseases in stages I–III.

For stage I patients increasingly a stereotactic approach is chosen, whereas for locoregionally advanced stages more fractionated forms of radiotherapy are the cornerstone of treatments, often combined with chemotherapy.

State of the art treatments for locoregionally advanced NSCLC comprise 60 Gy simultaneously applied to 2 cycles of chemotherapy. However, for toxicity reasons this approach is amenable only for about 30% of patients [1]. Furthermore, the premature closure of RTOG 0617 randomizing 60 Gy vs. 74 Gy suggests, that improvement of the results by intensifying radiation is improbable [2].

In contrast, sequential chemo-radiotherapeutic modalities seem to offer promising possibilities. A positive dose-response relationship in the range 60–100 Gy has been established for tumor control and survival [3]; and there is growing evidence that shortening of the overall treatment time is crucial [4,5]. However, until now radiation doses in escalation trials are always determined by dose constraints for normal tissues, not by features of tumors relevant for tumor control [3,6,7]. As a consequence, larger tumors in these trials are often treated with smaller doses than small tumors.

Recently, in our clinic the conformal target splitting technique has been developed for treating lung cancer, attended by a concept of rather tight margins in the treatment planning process [8-11]. Initially, high dose radiation therapies with conventional fractionation rendered encouraging results [12]. Thereafter, in order to lower treatment times, in a phase I/II trial up to 90 Gy to primary tumors and 63 Gy to nodes in 1.8 Gy bid fractions were applied, preceded by 2 cycles chemotherapy [13]. The results showed good tolerability and promising results for tumor control and survival. The here presented consecutive trial targets directly the doses required for tumor control in a differentiated mode. Radiation doses were related to tumor volume, treating larger tumors with higher doses. Primary endpoints are the local and regional tumor control rates at 2 years (as >90% of locoregional failures occur within 2 years). Secondary endpoints are survival and toxicity. With a minimum follow-up time of 2 years for patients alive, the final results of this prospective trial are presented.

## Methods and materials

### Trial design

Eligible were patients with nonresected, histologically/cytologically proven NSCLC in stages I through III B, and a Karnofsky Index =>60.

Radiotherapy had to be given twice daily with fractional doses of 1.8 Gy (ICRU Report 50), an interval of >10 h, at 5 days/week. Overall doses to the primary tumors were aligned along increasing tumor size within 4 groups in the range 73.8–90.0 Gy (Tables 1 and 2). The dose to macroscopically involved nodes had to be 54.0–72.0 Gy (adapted to the degree of extent and invasion, left to the discretion of the treating physician), to elective nodes 45.0 Gy within a region of about 6 cm cranial to macroscopically involved nodes. In stage I patients elective nodal irradiation could have been omitted.

**Table 1:** Patient (n = 160) and tumor (n = 164) characteristics


Age, years, median	66 (44–87)
Gender: m/f, n	119/41
Weightloss >5%/3 month, n (%)	38 (24)
Karnofsky Index, n (%)	
60	10 (6)
70	72/45)
80–100	78 (49)
Histology/cytology, n (%)	
Squamous cell carcinoma	104 (63)
Adenocarcinoma	43 (26)
NSC-n. o. s	17 (11)
AJCC-stage, n (%)	
I	38 (24)
II	6 (4)
III A	69 (43)
III B	47(29)
Affiliation according PT-Ø, n (%)	
Group 1 (< 2.5 cm)	27 (17)
Group 2 (2.5–4.5 cm)	94 (57)
Group 3 (4.5–6.0 cm)	30 (18)
Group 4 (> 6.0 cm)	13 (8)
Gross tumor volume (ccm, median/mean, range)	
Group 1	17/33 (2–167)
Group 2	50/68 (9–492)
Group 3	92/108 (50–205)
Group 4	163/238 (105–572)
Tumor localisation, n (%)	
Central	44 (27)
Peripheral	120 (73)

*Abbreviations*: NSC-n.o.s. = Non small cell-not otherwise specified; AJCC = American Joint Commission on Cancer, PT-Ø = Primary tumor diameter (mean number of 3 perpendicular diameters).

**Table 2:** Treatment characteristics


Total dose (Gy)	
Primary tumor	
Group 1	73.8
Group 2	79.2
Group 3	84.6
Group 4	90.0
Nodes (median, range)	59.4 (54.0–73.8)
Nodes electively*	45.0
Fractional dose (Gy)	1.8 bid
Interval	>10 h
Treatment duration (days, median, range)	33 (29–42)
Chemotherapy before radiotherapy (patients, %)	106 (66)
Cycles (n, range)	2 (1–6)

* to sites about 6 cm cranial to macroscopically involved nodes.

The number of cycles of induction chemotherapy was left to the discretion of the referring department; preferably it should have been limited to two cycles. The interval between chemotherapy and radiotherapy should have been < 8 days. Simultaneous chemotherapy was not allowed.

### Staging procedures

Staging evaluations included a medical history, physical examination, chest X-ray, bronchoscopy, 18-fluorodeoxyglucose-positron emission tomography (FTG-PET) and a CT-scan or MRI of the brain.

### Radiotherapy planning and delivery

Patients were set up in vacuum cradles, usually supine with the hands above the head. Planning CTs in treatment position were performed as ‘slow CTs’, with patients freely breathing (non-spiral CT; 4 s/slice; slice thickness 7 mm) or in a few patients as 4D-CT/average projection at the end of the accrual period (internal target volume concept, grossly summarizing and depicting the different positions of the moving tumor, rendering dispensable an extra-margin for tumor movement) [10]. A margin of 7 mm was added to the gross tumor volume (GTV) to draw the planning target volume (PTV). In general, pretherapeutic PET-CT scans were visually studied by the treating physician before drawing the PTV; only in cases of atelectasis a second, postchemotherapeutic PET-CT was performed and PET- and planning-CT scans were digitally matched. In patients receiving chemotherapy, the PTV was delineated at the postchemotherapy scans. PTVs of primary tumors and lymph nodes were drawn within the lung window and soft tissue window, respectively.

For patients with locoregionally advanced disease mostly the conformal target splitting technique was used [8,9].

Treatment plans were generated in a ‘forward’ planning process, minimizing especially radiation to healthy lung tissues, irrespective of its level or volume. In general the following lung dose constraints were applied: V20 (volume receiving >20 Gy) for a single lung 50%, V25 for both lungs 30%; in some patients these constraints were surpassed. A dose constraint for spinal cord was set at 45 Gy; and for esophagus at 80 Gy (measured in the center of the esophagus at its most exposed level). Examples of treatment plans have been published recently [9].

Treatments were applied by 15 MV photons.

The set-up of patients was adjusted in 3 dimensions before every treatment, matching anatomical structures as esophagus, trachea, main bronchi by means of two kv-images in two dimensions [14].

### Medicinal agents

Chemotherapeutically, in general cisplatin or carboplatin containing doublets were used.

If parts of the esophagus were within or near the PTV, an antimycotic prophylaxis was given (Amphotericine B lozengers, 4 times daily, during the full course of radiotherapy) [15,16].

### Follow-up procedures

Patients were seen for assessment of toxicity and tumor control 6 and 12 weeks after the end of radiotherapy, then every 3 months for the first year, every 4 months during the second and third year, hence every six months. At the first control a chest X-ray, at all other controls thoracic CT-scans were performed. Local or regional tumor progression was diagnosed, if there was an increase in tumor volume compared with the previous CT-scan. In case of doubt a FDG-PET had to be performed.

Acute and late toxicity was scored according to the RTOG/EORTC criteria except for pulmonary toxicity grade 1, because the criterion ‘mild symptoms of dry cough or dyspnea on exertion’ is common in these pulmonary compromised patients. As in our experience in rare cases pneumonitis as an acute side effect can be present until 6 months after therapy, toxicity is considered late if it persisted or developed beyond 6 months after the completion of radiotherapy.

### Statistical analysis

Overall survival and local tumor control rates were calculated using the Kaplan-Meier method. All time intervals refer to the start of therapy, induction chemotherapy included. The median follow up time for all patients is 26.0 months (3.8–96), for survivors 38.1 months (24.8–96 months). No patient has been lost to follow-up.

In order to collect sufficient data in the four treatment groups, it was prospectively determined to enrol at least 150 patients in this trial.

The study was performed with consent of the medical ethics committee of the province Salzburg. All patients gave informed consent.

## Results

### Patient and treatment parameters

From January 2004 until December 2009 160 patients with 164 histologically/cytologically proven NSCLC were enrolled. This corresponds to 96% of all NSCLC patients in stages I–IIIB referred to our department in this period (patients with malignant pleural effusions, pancoast tumors and one patient with pulmonal fibrosis excluded, see below). Five of the 7 patients not enrolled presented with KI <60%, two patients refused to be treated twice daily for geographical reasons. Hence we consider the study population as continuously referred and unselected.

Patient and tumor characteristics are shown in Table 1. Notably, 24% presented with >5% weight loss during the three months preceding diagnosis; and 51% had a KI <80%.

Treatment parameters can be seen in Table 2. All but five primary tumors received the dose corresponding to the respective treatment group (in 3 patients of group 2 and in 2 patients of group 3, doses were lowered for 3 fractions; for dose/volume issues or a reduced general status of the patients). The vast majority of stage II/III patients received an elective nodal treatment cranial to macroscopically involved sites. For dose-volume reasons in 18 patients (15%) this elective treatment was not performed.

### Local and regional tumor control, distant failures

**Figure 1:**
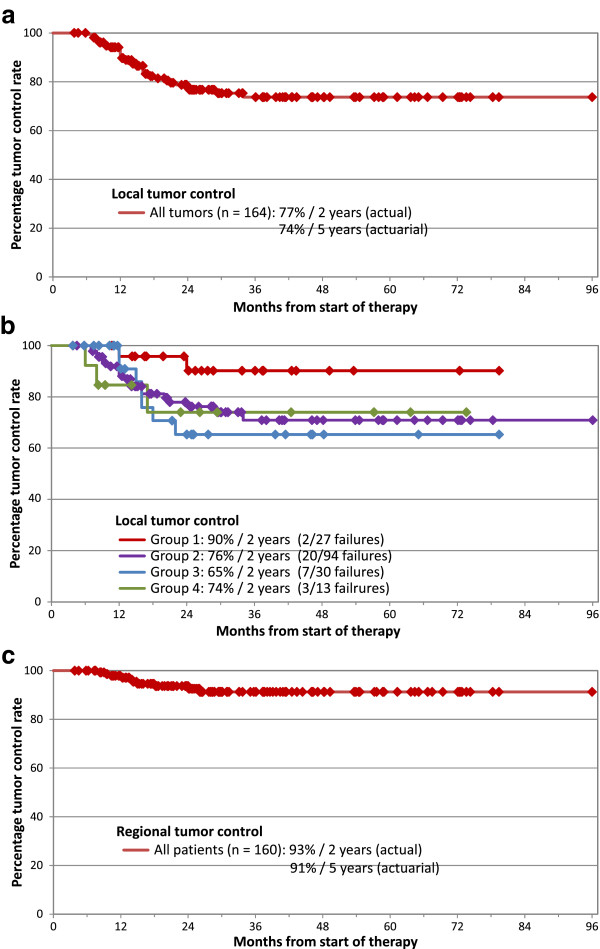
Kaplan-Maier plots for a) local tumor control all tumors; b) local tumor control by tumor size groups; c) regional tumor control all patients.

We observed 32 local failures, 30 of them occurred within 2 years; this results in an actual 2- and actuarial 5-year local tumor control rate of 77% and 74%, respectively (Figure 1a). The distribution of local recurrences within the four groups can be seen in Figure 1b. The best results show the patients in group 1 (90% tumor control at 2 years), the control rates in group 2 to 4 are similar and lie between 65% and 76%/2 years.

10 patients showed an isolated regional failure (without a local recurrence) at a median time of 14 months (range 8–26 months), corresponding to a regional control rate of 93% at 2 years (Figure 1c). Five of these failed at distant sites simultaneously. In 6 patients the regional failure was located in originally irradiated sites (54.0–63.0 Gy), in 4 patients in non-treated regions (three of these four patients belong to the small group of 18 patients, where an elective nodal treatment primarily was omitted).

In 57 patients (36%) hematogenous metastases appeared during the course of the disease, mostly in the brain (23 patients).

### Overall survival

**Figure 2:**
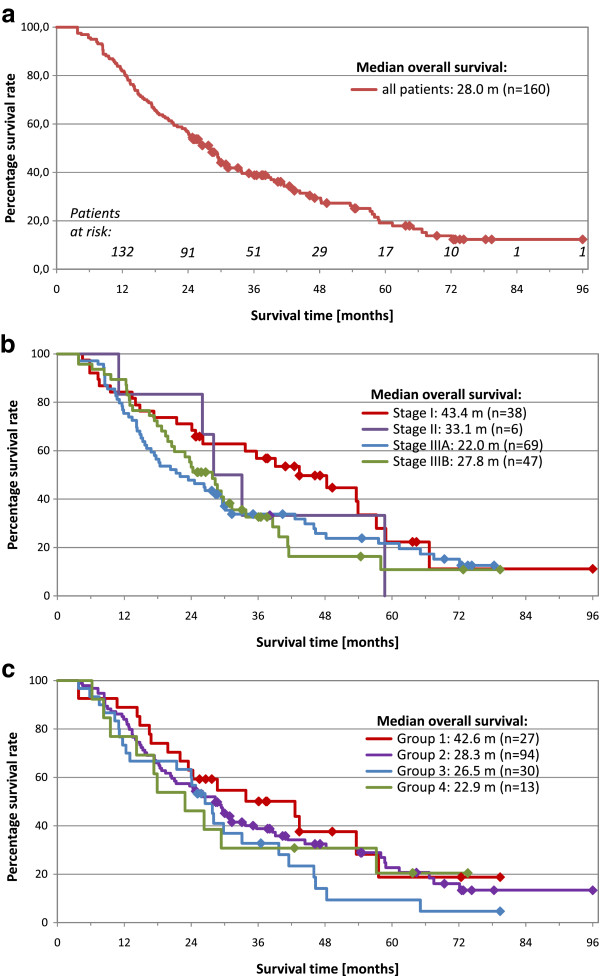
**Overall survival.** Kaplan-Maier plots for **a**) all patients; **b**) patients by tumor stage; **c**) patients by tumor size groups.

The actual overall 2 year-survival rate for all patients and the actuarial 5 year-survival rate is 57% and 19%, respectively, the median survival time 28.0 months (Figure 2a). The overall median and 5 year-survival rate for patients in stage I, II, IIIA, IIIB, IIIA + B amounts to 43.4, 33.1, 22.0, 27.8, 24.3 months and to 22%, 33%, 22%, 11%, 18%, respectively (Figures 2b). Survival curves according to tumor size groups are depicted in Figure 2c.

30 patients died from intercurrent diseases at a median time of 15.3 months (range 3.8–72.1 months). Nine of them have had stage I tumors and were referred to primary RT due to a low performance status.

### Toxicity

Table 3 shows the non-hematologic toxicities. In two patients a treatment-related death occurred: progressive pulmonary fibrosis (not pneumonitis) 5 and 6 months after the end of radiotherapy. The patients were treated for stage IIIA tumors, with unexceptional pulmonary doses (V25 30% and 23%, respectively). In the pretherapeutic CT scans of both patients clear radiologic signs of existing pulmonary fibrosis were visible. These events occurred within 2 months during the second year of the accrual period. Thereafter patients with pulmonary fibrosis were excluded from enrolment in this trial. Apart from that, treatments were well tolerated.

**Table 3:** Acute (A) and late (B) non-hematologic toxicity according to EORTC/RTOG criteria, n (%)

		**Grade 1**	**Grade 2**	**Grade 3**	**Grade 4**	**Grade 5**
A	Esophagus	51 (32)	16 (10)	7 (4)	-	-
	Lung	NA	10 (7)	6 (4)	-	2 (1)
B	Esophagus	-	-	1 (1)	-	-
	Lung	NA	15 (9)	-	-	-

Abbreviations: EORTC/RTOG = European Organisation for Research and Treatment of Cancer/Radiation Therapy Oncology Group.; NA = not assessed.

One patient showed late esophageal toxicity grade 3 (stenosis, 7 months after the end of radiotherapy with moderate esophageal doses, treated by stenting; a few months later the patient died for a local recurrence). Almost all patients present slight to moderate posttherapeutic densities in the lung parenchyma, which did not cause symptoms > grade 2.

Four patients died from a pulmonary hemorrhage. In three of them a recurring central tumor was diagnosed before. The fourth patient, treated with 79.2 Gy for a central tumor with close proximity to the bronchial arteries for about 4 cm, died 6.5 months after finishing radiotherapy. The autopsy demonstrated a leakage of a bronchial artery without detection of a recurrent tumor, nor necrotic tissue. The most probable cause of death of this patient is a brochoarterial fistula arisen after retraction of the central, infiltrating tumor.

## Discussion

We consider the concept and mode of implementation of DART-bid a novel approach for treating non-resected NSCLC. Usually in dose escalation protocols, for reasons of tolerability, doses are determined by dose constraints for normal tissues, not by features relevant for tumor control as e.g. tumor size [3,6,7,17-19]. As a consequence large tumors are often treated with smaller doses than small tumors and presumably high volume tumors are occasionally excluded from enrolment in protocols. DART-bid targets straightforward the doses required for a high level of tumor control. New is also the differentiation of doses on tumor sites: it is sufficient to treat nodes with lower doses compared to primary tumors, because generally nodes are well oxygenized, without necrotic areas and of smaller size.

Table 4 shows that in our series the results compare favourably with the outcome of other modalities in all endpoints: local and regional tumor control, survival, toxicity.

**Table 4:** Selected trials in the treatment of NSCLC

	**Author**	**n**	**Stages**	**Selection of study population**	**Treatment**	**Esoph. toxicity ≥gr.3 (%)**	**Tumor control rate at 2 y.: Local/locoreg.**	**Overall survival**	
								**Median (mo)**	**2/5 y (%)**
A	Curran et al. [1]	195	II, III	good PS	2 × Ch, sequ 60 Gy	4	NR	14.6	-/10
		195	II, III	good PS	2 × Ch, sim 60 Gy	23	NR	17 (p = 0.046)	-/16
		187	II, III	good PS	2 × Ch, sim 69.6 Gy/1.2 Gy bid	45	NR	16	-/13
	Furuse et al. [20]	158	III	---	2 × Ch, sequ 56 Gy	2	NR	13.3	27/9
		156	III	---	2 × Ch, sim 56 Gy split course	3	NR	16.5 (p = 0.04)	35/16
	Fournel et al. [21]	101	III	good PS	3 × Ch, sequ 66 Gy	3	NR	14.5	26/14(4y)
		100	III	good PS	2 × Ch, sim 66 Gy, plus 2 × Ch.	32	NR	16.3 (p = 0,24)	39/21(4y)
B	Stinchcombe et al. [25]	62	III	good PS, V20 < 35%	Ind Ch 2×, 60–74 Gy sim Ch	8	NR	25	-/27
	Bradley et al. [26]	53	I–III	good PS, V20 < 30%	74 Gy, sim Ch weekly	12	NR	25.9	NR
C	Kong et al. [3]	106	I–III	unselected, long accrual period	63–102.9 Gy/2.1 Gy/6–10w.; 19% ind Ch 2×	7	-/40	19.0	37/13
	Bradley et al. [6]	177	I–III	44% stage I, V20 <37%	70.9–90.3 Gy/2.15 Gy/6.5–8,5 w.; 14% ind Ch	3	---	NR	NR
	Wurstbauer et al. [12]	124	I–III	unselected	80–96 Gy primary t., 69.3 Gy nodes/2.0–2.2 Gy/8–9 w., 47% ind Ch	0	-/49	19.6	39/11
D	Van Baardwijk et al. [7]	166	I–III	unselected	64.8 Gy (50.5–79.2)/1.8 Gy bid, i = 8 h/4.5 w.; 55% ind. Ch 3×	5	NR	21.0	45/--
	Saunders et al. [31]	225	I–III	good PS	60 Gy/2 Gy/6 weeks	3	NR	13	21/NR
		338	I–III	good PS	54 Gy/1.5 Gy tid/12d/i ≥ 6 h	19	NR	16.5 (p = 0.008)	30/NR
	Wurstbauer et al. [13]	30	I–III	unselected	84.6 Gy primary t., 63 Gy nodes/1.8 Gy bid, i >10 h/5 w.; 63% ind. Ch 2×	7	--/61	27.7	63/23
	Current study	160	I–III	unselected	73.8–90.0 Gy primary t., 59.4 Gy nodes/1.8 Gy bid, i >10 h/5 w.; 66% ind. Ch 2×	4	77/70	28.0	57/19
		(116	III A + B					24.3	51/18)

A. Randomized studies comparing sequential versus simultaneous radio-/chemotherapy. B. Further simultaneous studies. C. Radiation dose escalation trials. D. Trials employing accelerated radiotherapy.

*Abbreviation*s: NR = not reported; Ch = chemotherapy; sequ = sequential, sim = simultaneous, ind = induction, PS = Performance status.

### Local tumor control

In reported studies of simultaneous radio-/chemotherapies and dose escalation protocols locoregional tumor control is mostly not appropriately examined [1,3,6,20,21]. Corresponding diagnostic methods and intervals are not prospectively performed. Often only the patterns of first relapses are reported. Simultaneous radio-/chemotherapies seem to achieve definitive local tumor control in about 30–40% of the patients [1,20,21], sequential high-dose treatments with conventional fractionation in about 40–50% [12,22].

Total dose levels in our protocol were set with the intention of a high degree of tumor control, according to our experience in the preceding phase I/II trial [13]. A control rate of 90% for the small tumors (group 1) and between 65% and 76% for the larger ones (group 2–4) seems to be impressive, although there is still space for improvement. In our patients, image guidance was performed in every fraction, matching anatomical structures as trachea, main bronchi, esophagus via two orthogonal kv-images. Recently, in a series of 30 patients we compared the topographic accuracy matching anatomical structures as described versus matching on gold fiducials, implanted bronchoscopically in or near the primary tumor. In 8 out of 30 patients (27%) in >30% of fractions the vector of the position corrected via fiducials exceeded 7 mm, which is our commonly used margin from GTV to PTV. In other words, without position correction along fiducials, in a quarter of the patients in more than a third of the fractions parts of the GTV come to lie outside the treated volume. Hence, a further improvement of local control might not primarily be a matter of raising the total dose, but of raising geographic accuracy. Therefore, in our successive series of NSCLC patients, tending to further improve local tumor control, total doses remain unchanged and IGRT with gold fiducials (or cone beam CT) is performed routinely.

It has been discussed, if local tumor control should be adjudged only if tumors initially respond with a partial or complete response radiologically (RECIST-criteria) [23]. We cannot share this opinion, because occasionally we observed lung tumors presenting at CT-controls as opacities in dimensions of a minor response for periods longer than 10 years.

### Regional tumor control

The regional tumor control rate of 93% in our study is high and confirms the concept to apply lower doses to nodes in comparison to primary tumors.

Regarding elective nodal irradiation, in four patients an isolated nodal recurrence in previously untreated sites occurred. Only one of these patients belong to the group of 104 stage II/III patients treated primarily electively cranial to macroscopically involved nodes, whereas three belong to the small group of 18 patients, in which an elective nodal irradiation was postponed for volume issues. This confirms the necessity of elective nodal radiation cranial to involved nodes. In general nowadays, elective nodal irradiation has been abandoned, in order to gain potential to raise the dose to macroscopic tumor; but series conducted in this way report isolated elective nodal recurrences in up to 9% of the patients [6,24].

### Survival

A median overall survival time of 28.0 months for all patients and 24.3 months for stage III patients compares favourably with the results of other approaches (Table 4). Simultaneous chemo-/radiotherapies result in survival times of about 17 months [1,20,21] (Table 4). The patients in these studies were not PET staged and therefore an imbalance for stage migration may occur; however, in contrast to our unselected population, these patients were in good general condition, without considerable comorbidities and without weight loss. There are simultaneous treatments applying 74 Gy, achieving survival times up to 26 months; in these series the additional selection criterion of a relatively strict V20 of 30–35% is used, most probably a surrogate for patients with lower ‘tumor load’ [25,26].

### Toxicity

Except the two patients with pre-existing pulmonary fibrosis (see above), treatments were tolerated very well. We attribute the good tolerability and the fact, that 97% of the primary tumors could have been treated with the dose of the respective group, to three reasons: the beam arrangements inherent to the target splitting technique, the use of rather tight margins and the differentiation of doses [8-10]. These issues have been discussed in detail in the report of the preceding phase I/II trial [13]. Briefly, target splitting is a technique with a high potential of sparing organs at risk. Concerning the tight margin of only 7 mm from GTV to PTV: the internal target volume concept, using slow planning CTs or average mode 4D planning CTs, averts the necessity of adding a general extra margin for tumor motion (internal margin). And, as in stereotactic radiotherapy, we consider a margin for microscopic spread from GTV to a clinical target volume (CTV) in high dose radiotherapy dispensable, because a sufficient dose to the rim of microscopic disease (about 45 Gy in 2,5 weeks) is delivered anyway.

### Simultaneous vs. sequential therapies with intensified radiation component

At present simultaneous chemo-/radiotherapies are considered state of the art for locoregionally advanced NSCLC. Several randomized trials have proven an advantage of 60 Gy applied simultaneously vs. sequentially [1,20,21]. The radiosensitizing chemotherapeutic effect improves the local tumor control, median overall survival times raise from about 14 to 17 months. As also side effects act simultaneously, these therapies are more toxic; therefore only about 30% of stage III patients are amenable for this approach [1]. A further, grave drawback of the simultaneous approach is the lack of improvements raising radiation doses. RTOG 0617, randomizing 60 Gy vs. 74 Gy recently was closed prematurely. An interim analysis showed that 74 Gy could not produce a survival benefit and possibly give worse results [2]. Final analysis must be expected, but 60 Gy, applied with 3D-conformal and IMRT techniques, seem to be the upper dose limit in simultaneous radio-/chemotherapies.

In contrast, sequential therapies with intensified radiation components may offer promising developments.

The potential of 2 cycles induction chemotherapy to eradicate peripheral micrometastases, thus lowering the incidence of later distant relapse, has been proven in trials [27,28] (and often induction chemotherapies also provide an advantegous shrinkage of tumors before starting radiotherapy). However, as discussd in detail in our phase I/II trial report, fast repopulation of tumor cells between cycles of chemotherapy and during the interval between chemotherapy and radiotherapy might occur [13,29,30].To prevent this, we avoid to apply more than 2 cycles chemotherapy, and try to keep the interval to radiotherapy <8 days.

Similarly, a short overall radiation treatment time is crucial [5]. Fowler estimates a loss of local tumor control of 11% per week for treatment times beyond 4 weeks [4].This reflects the drawback of dose escalation protocols performed with conventional fractionation [3,6,12] (Table 4). One way to lower radiation treatment time is treating patients more often than once daily [7,13,31] (Table 4). Van Baardwijk et al. report on 1.8 Gy bid treatments with an individualized prescription based on normal tissue dose constraints (19 Gy mean lung dose) [7]. A median dose of 64.8 Gy (range 50.4–79.2 Gy) was applied and a median overall survival time of 21.0 months was achieved. Equal doses to primary tumors and nodes were given. Treatments were safe, figures for locoregional tumor control are not reported. DART-bid enables the application of higher doses; and regarding the low toxicity, these doses could even be raised, if necessary. The latter is investigated in ongoing studies, in the mode described above.

Another method to lower treatment times is hypofractionation. Mature result of such regimes are not yet available. Substantial side effects, especially concerning central tumors, could be an issue in this setting.

## Conclusions

DART-bid following induction chemotherapy yields a high level of locoregional tumor control and survival times. In general it is well tolerated. In all outcome parameters it seems to compare favourably with simultaneous chemo-radiotherapies, while being additionally amenable for an unselected patient population.

## Competing interests

The authors declare that they have no competing interests.

## Authors’ contributions

KW designed the study, collected the data and drafted the manuscript. HD developed the various specific techniques employed. KW, KD, PK, FZ, BL, PP, BW, MS and FS treated the patients. All authors read and approved the final manuscript.
